# Visualization and Analysis of the Mapping Knowledge Domain of Acupuncture and Central Nervous System Cell Apoptosis

**DOI:** 10.1155/2022/1751702

**Published:** 2022-04-14

**Authors:** Rongming Qi, Zhe Xue, Yaru Liu

**Affiliations:** ^1^School of Acupuncture-Moxibustion and Tuina, Beijing University of Chinese Medicine, Beijing 100029, China; ^2^School of Traditional Chinese Medicine Beijing University of Chinese Medicine, Beijing 100029, China

## Abstract

Chinese acupuncture therapy has demonstrated good clinical effects on neurological diseases and is widely used internationally. In the past 20 years, an increasing number of researchers around the world have devoted themselves to the study of the effect and mechanism of acupuncture for the treatment of central nervous system cell apoptosis. To discover the current research status of acupuncture-induced antiapoptosis in the central nervous system, we used the method of scientometric research and data visualization software to visually analyse 155 articles. The findings are as follows. First, the antiapoptosis effects of acupuncture in the central nervous system have received increasing attention overseas and domestically. China and the United States have leading positions in this research field. Second, 5 stable and high-yielding research teams have been formed in the field of acupuncture-induced antiapoptosis. The main research directions of these teams are electroacupuncture (EA) pretreatment for the central nervous system cell apoptosis, acupuncture for antineuronal apoptosis in vascular dementia, EA regulation of related signalling pathways, EA regulation of nerve cell apoptosis and autophagy after stroke, and EA regulation of the MAPK signalling pathway. Researchers on teams with more extensive cooperation have more research results and better research continuity. Third, there are diversified research hotspots. The original research hotspots are still receiving attention, and new hotspots have emerged in recent years.

## 1. Introduction

Apoptosis is a process of programmed cell death that occurs in multicellular organisms [1]. During this process, cells undergo a series of changes, including blebbing, shrinkage, nuclear fragmentation, chromatin condensation, chromosomal DNA fragmentation, and global mRNA decay. Apoptosis plays an important role in neurodegenerative diseases, including cerebral ischaemia–reperfusion injury, Alzheimer's disease (AD), spinal cord injury (SCI), depression, stress, Parkinson's disease (PD), peripheral nerve injury, and intracerebral haemorrhage (ICH) [2].

Acupuncture has been used as an economical treatment with few adverse effects in China for more than 2000 years. To date, modern research has shown that acupuncture can protect nerve tissue from damage by inhibiting excessive cell apoptosis and that acupuncture has few side effects, especially for patients with neurological diseases [3, 4]. Therefore, in the past two decades, research on the effect and mechanism of acupuncture on neuronal cell apoptosis has received increasing attention. This research field has accumulated a large amount of literature. A timely review of the previous literature will help researchers understand the current research status in this field and will provide inspiration for new research directions in the future.

Our study used bibliometric research methods and data visualization software to comprehensively and intuitively present the current research status and hotspots in the field of acupuncture-induced antiapoptosis of central nervous system cells to readers. The specific content will be explained from two aspects.

First, this study aimed to show the temporal and spatial distribution of researchers, countries and regions, institutions, and journals in the field of acupuncture-induced antiapoptosis in the central nervous system. Moreover, our study mainly focused on the display and analysis of the internal collaboration relationship of each research resource. Second, to discover research hotspots and research trends, we drew a cooccurrence map of keywords in the field of acupuncture and moxibustion against central nervous system cell apoptosis and performed a cluster analysis of the main keywords. This article is presented in accordance with the following content: methods, literature analysis, conclusions, and future prospects.

## 2. Materials and Methods

### 2.1. Data Source

The literature was retrieved from Web of Science (WOS), the scope of which was the WOS core collection, and the search strategy was TS = ((acupuncture OR electroacupuncture OR body acupuncture OR scalp acupuncture OR auricular acupuncture OR wrist-ankle acupuncture OR Warm Acupuncture OR Acupuncture Points OR Acupuncture Therapy OR Manual Acupuncture OR Abdominal Acupuncture OR acupoint injection OR needle OR needling) AND (cerebral apoptosis OR brain apoptosis OR nerve apoptosis OR nerve cell apoptosis)). The search dates ranged from 1985 to 2021. The retrieval time was 21 : 10 PM on October 27, 2021. A total of 232 articles were retrieved. A total of 155 documents were finally obtained by eliminating irrelevant documents and duplicate documents. The whole retrieval process was completed at 23 : 37 PM on October 27, 2021. The retrieved documents were exported in plain text format and in the form of “complete records and cited references.”

### 2.2. Analysis Method and Tools

The analysis methods used in this study were cocitation analysis and cooccurrence analysis. In cocitation analysis, when two (or more) papers are cited by one or more subsequent papers at the same time, the two papers are said to have a cocitation relationship. In cooccurrence analysis, “cooccurrence” refers to the phenomenon when the characteristic item information of documents is similar. Features include external and internal features of the literature, such as the title, author, keywords, and organization. “Cooccurrence analysis” is a quantitative study of cooccurrence to reveal the content relevance of information and the knowledge implied by the feature items. We used CiteSpace 5.8. R1 as the analysis tool.

## 3. Results

### 3.1. Analysis of Annual Publications

**Figure 1:**
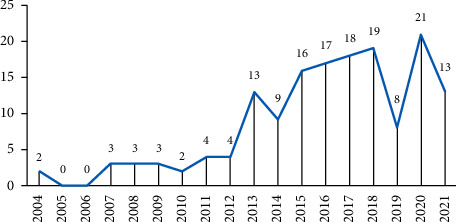
Annual number of papers published in acupuncture and central nervous system cell apoptosis, 2004–2021. Note: the data in 2021 are still being updated.

Figure 1 is a graph of the annual number of articles published. The value marked at each point on the curve represents the number of papers published that year. The first paper in this research field was published in 2004, and to date, 155 articles have been published. Over the past 18 years, the annual publication volume in this field has shown an overall upwards trend. We divided the research enthusiasm in the field of acupuncture and antineuronal apoptosis into two stages according to the annual publication volume change trend.

  Initial stage: before 2012, the volume of annual publications remained at a relatively low level.
  Growth stage: from 2013 to 2021, there was a significant increase in the number of publications. Although there were two troughs in 2014 and 2019, the overall trend was an increase.

### 3.2. Countries and Regions

**Figure 2:**
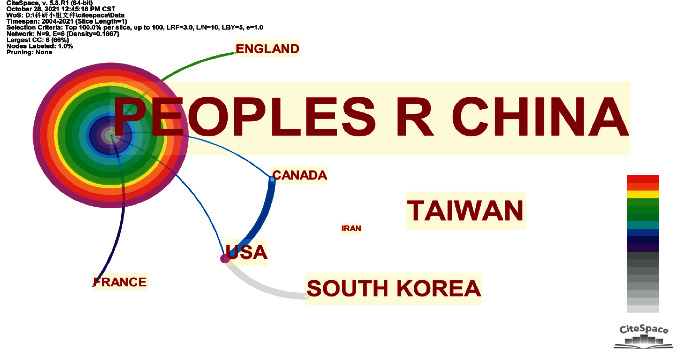
Research countries and regions in acupuncture and antineuronal apoptosis.

Figure 2 intuitively shows the participation and cooperation of various countries and regions in the field of acupuncture and antineuronal apoptosis research. The dots of different sizes in the picture represent different countries or regions, and their names are marked. The size of the point is positively correlated with the amount of research literature published in the country and region. The connecting lines between points represent the cooperation between different countries and regions. The thickness of the connection is positively correlated with the frequency of cooperation. Each point is composed of multiple concentric rings of different colours. In addition, the colour of the connection is different. The colder the colour of the dots and lines, the longer since the first occurrence, and the warmer the colour, the more recent the occurrence. The outermost purple circle represents intermediary centrality. The thicker the purple ring, the higher its centrality.

There are 9 countries and regions participating in acupuncture and antineuronal apoptosis research (Table 1). The top 4 countries and regions in terms of publication volume are China, Taiwan, South Korea, and the United States. Among them, China issued 137 documents, accounting for 88.39% of the total issued documents. Intermediary centrality represents the strength of the connection with other countries and regions. The greater the intermediary centrality of a country or region, the wider the cooperation with other countries and regions, and the stronger the communication and cohesion. The intermediary centrality of China and the United States in this field is 0.5 and 0.29, respectively, ranking as the top two countries. China and the United States are in a leading position in this research field. In Table 1, “Year” represents the year when the first literature was published in each country and region. France, Egypt, the United Kingdom, and Iran have also published their first research articles in this field in recent years in 2013, 2015, 2018, and 2019, respectively. This shows that an increasing number of countries and regions have begun to pay attention to acupuncture and antineuronal apoptosis.

Figure 2 shows that only a relatively stable cooperative network has been formed in the field of acupuncture and antineuronal apoptosis research. The network includes China and the United States, two countries with high centrality. Research in the United States was carried out very early; thus, international cooperation is more extensive. Only 4 papers have been published in the United States, but they are highly intermediary. The intensity of cooperation between the United States and other countries and regions is greater than China's international cooperation. Although the number of publications in Taiwan Province of China and South Korea is small, they have made progress. However, Taiwan's research lacks international cooperation.

### 3.3. Research Organizations

**Figure 3:**
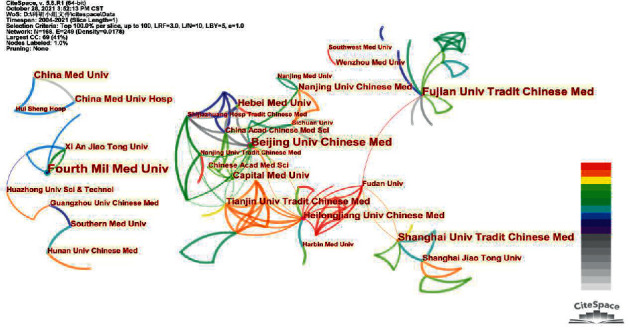
Institutions involved in the study of acupuncture for the treatment of central nervous system cell apoptosis.

Figure 3 shows the institutional cooperation networks. Dots of different sizes represent different institutions and are labelled with the institutions' names. The sizes of dots and texts are positively correlated with the amount of research literature published by the institution. The connecting lines between the points represent cooperation between agencies. The thickness of the connection is positively correlated with the frequency of cooperation. The colours of the connecting wires are also different. The colder the point and line colours are, the longer ago they first appeared, and the warmer the colours are, the more recently they appeared. There are five major institutional cooperation networks in total.

The largest network is in the middle of the image. This network has the most different colours and the most lines. This indicates that the cooperation between institutions is the closest, the research started earlier, and the research has lasted the longest. The network is centred on Beijing University of Chinese Medicine, Tianjin University of Traditional Chinese Medicine, Capital Medical University, Hebei University of Traditional Chinese Medicine, Tianjin University of Traditional Chinese Medicine, and Heilongjiang University of Traditional Chinese Medicine. The China Academy of Traditional Chinese Medicine has also made great contributions. The main institutions in the cooperation network are distributed in an area with Beijing as the centre and a radius of 300 km. Particular attention should be given to the small red and yellow network, with Heilongjiang University of Traditional Chinese Medicine as the key connection point in this huge cooperation network. This shows that Heilongjiang University of Traditional Chinese Medicine is a relatively active research institution in the field of acupuncture and antineuronal apoptosis in recent years. Heilongjiang University of Traditional Chinese Medicine cooperates with Beijing University of Chinese Medicine, Tianjin University of Traditional Chinese Medicine, Fudan University, Harbin Medical University, and other research institutions.

The right side of the picture is a cooperation network centred on the Fourth Military Medical University. Most of the institutions in the network are located in Western and South China. The colours of the cooperative network range from blue to orange, indicating that their work began early and continues to this day. The Fourth Military Medical University has an international cooperation with Stony Brook University (SUNY) in the United States. SUNY is one of the few organizations that carry out international cooperation in the field of acupuncture and antineuronal apoptosis. The network is dominated by cool colours, indicating that the cooperation between institutions took place earlier. However, these institutions have not cooperated in recent years.

The network centred on Shanghai University of Chinese Medicine and Shanghai Jiao Tong University is located in the lower right corner of the picture. They started a study in recent years and are following up. In addition, Shanghai University of Traditional Chinese Medicine and Fudan University have recently started collaborative research. The main colour of the cooperation network centred on Fujian University of Traditional Chinese Medicine is green. Fujian University of Traditional Chinese Medicine is located in the upper right corner of the picture. This shows that interagency cooperation has occurred in recent years. Fujian University of Traditional Chinese Medicine also collaborated with Fudan University. These two networks show a tendency to integrate into the larger network.

By analysing the picture information, we find that the cooperation between institutions is breaking the space constraints. The early cooperation between institutions shows a significant spatial clustering feature. Neighbouring institutions can more frequently cooperate. However, the distance between cooperative institutions has often been relatively far in recent years. Furthermore, there are many high publication volume research institutions in the largest cooperative network, such as Beijing University of Chinese Medicine, Tianjin University of Traditional Chinese Medicine, and Capital Medical University. This shows that cooperation is an important factor in promoting the output of research results. Moreover, cooperative research on acupuncture and antineuronal apoptosis is mostly carried out between scientific research institutions in China. There is a lack of extensive and long-term international cooperation.

### 3.4. Main Authors' Cooperation Networks

**Figure 4:**
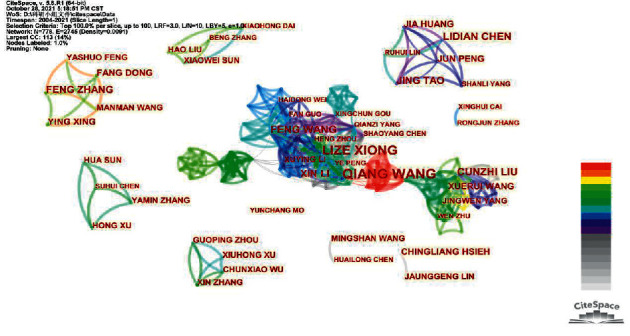
Cooperative network of authors involved in the study of acupuncture for the treatment of central nervous system cell apoptosis.

Since the first paper was published on January 9, 2004, a total of 778 researchers have participated in the publication of papers in this field.  Figure 4 shows the cooperative network of authors with more than 3 publications. Each dot represents an author, and the larger the dot is, the more articles that author has published. The connections between dots indicate collaborative relationships between two authors. The more times they cooperate, the stronger the connection is. The colour of the line is related to the time of cooperation. The warmer the colour is, the more recently the collaboration happened, and the colder the colour is, the longer ago the collaboration happened.  Figure 4 shows that five stable and high-yielding research teams have formed in the field of acupuncture for the treatment of central nervous system cell apoptosis.

Table 2 shows the representative authors and main research areas of these 5 research teams.

Qiang Wang and Cunzhi Liu are in the same large cooperative network and are in different study groups. They play an important role in the cooperative network. Qiang Wang's team believes that electroacupuncture preconditioning can be used as a new way to protect against cerebral ischaemia–reperfusion injury [5]. They found that electroacupuncture preconditioning could protect nerve cells in rats with cerebral ischaemia by regulating N-Myc downstream-regulated gene 2, *α*7nAChR activation, and the endocannabinoid system [6–12].

Cunzhi Liu's team focuses on the neuroprotective effects of acupuncture on rats with vascular dementia. They found that acupuncture reduces neuronal apoptosis in rats with vascular dementia by regulating oxidative stress in the rat hippocampus, NF-kB activation, Ref-1 expression, and mitochondrial function [13–18].

Lidian Chen's team has discovered in recent years that electroacupuncture can regulate multiple signal pathways to exert an antiapoptotic effect on nerve cells, for example, in peri-ischaemic regions, by modulating the p38, extracellular signal-regulated kinase (ERK1/2), c-Jun N-terminal kinase (JNK), and PI3K/Akt signalling pathways. In addition, EA markedly activated the cyclic adenosine monophosphate (cAMP) response element-binding protein (CREB) signalling pathway, resulting in the inhibition of cerebral cell apoptosis in the ischaemic penumbra [19–23].

Feng Zhang's team discovered that the PTEN signalling pathway, PI3K/AKT pathway, and ERK/JNK/p38 signalling pathway are key pathways by which EA inhibits neuronal apoptosis after stroke [24–27].

Chunxiao Wu's team focuses on EA to reduce neuronal apoptosis in animals with cerebral ischaemia–reperfusion injury by regulating the mitogen-activated protein kinase (MAPK) pathway. Their research involves multiple subpathways in the MAPK pathway, such as ERK, P38, and JNK [28–31].

By calculating the burst value of the publications of each data node, we can observe the sudden change in the publications of each node. This number has positive implications for our understanding of key researchers and key time periods in the field. Among all the researchers, only Qiang Wang, Lize Xiong, and Xin Li have burst values (Table 3). Their burst values appear in 2009, 2009, and 2011. In the corresponding time period, the published instruments of the three authors increased significantly. To date, Qiang Wang, Lize Xiong, and Xin Li have published 16, 13, and 6 papers, respectively. They are currently the three most prolific researchers in the field of acupuncture and moxibustion for the treatment of central nervous system cell apoptosis, and they are on the same research team. Thus, the more extensive the cooperation, the better the continuity of the research and the richer the research results. Academic exchange plays a positive role in promoting scientific research.

### 3.5. Main Journals

Journals are an important platform for academic exchanges and dissemination. The number of journal publications can reflect the degree of interest in a particular research field. To date, a total of 951 journals have participated in the publication of research papers about acupuncture and antineuronal apoptosis. We selected the leading journals in this field according to the citation volume. There are 10 journals with more than 50 citations (Table 4). Stroke had the most citations (110), followed by Brain Res and Neurosci Lett, with 94 and 91 citations, respectively. The citation quantity of a journal can indirectly reflect the academic value of the journal. These results show that these journals have high academic value.

We choose the top five cited journals for our analysis. Taking them as the focus of the analysis, we can discuss the research hotspots in this field. The top five cited journals are Stroke, Brain Research, Neuroscience Letters, PLOS One, Journal of Cerebral Blood Flow, and Metabolism.

The main points of the articles published in Stroke are as follows. Improvement in cognition and hippocampal synaptic plasticity induced by acupuncture is achieved via the activation of D1/D5 receptors in 2VO rats [32]. Pretreatment with EA increases the production of the endocannabinoid 2-arachidonylglycerol and N-arachidonoylethanolamine, which elicits protective effects against transient cerebral ischaemia through CB1 receptors. These mechanisms may lead to rapid tolerance to focal cerebral ischaemia [8].

The research results included in Brain Research are mainly concentrated in the following aspects. The effectiveness of EA preconditioning against HIBI may be mediated via the opening of KATP channels [33]. EA may inhibit autophagy in the hippocampus by reducing *β*-catenin/COX-2 protein expression and effectively alleviating central poststroke pain (CPSP) [34]. EA treatment could be a neuroprotective therapy for the cognitive dysfunction induced by AMIR events. It may be related to balancing the autonomic nervous system, inhibiting neuronal apoptosis, hindering the activation of microglia, reducing oxidative stress, and inhibiting central and peripheral inflammatory responses [35]. EA pretreatment enhances STAT3 activation via CB1R to protect against cerebral ischaemia, suggesting that STAT3 activation may be a novel target for stroke intervention [10]. EA pretreatment may promote spinal I/R injury through the inhibition of HMGB1 release in a LXA4 receptor-dependent manner [36]. LI/R can result in cognitive dysfunction related to activated microglia and elevated oxidative stress, and EA has neuroprotective potential mediated, at least in part, by the inhibition of microglial activation and the attenuation of oxidative stress [37]. The formation of an SDF-1*α* concentration gradient can induce the mobilization and homing of EPCs. EA can accelerate and increase the formation of the SDF-1*α* concentration gradient to further induce the mobilization of EPCs and angiogenesis in the ischaemic brain and improve neurological function recovery [38].

The main points of articles included in Neuroscience Letters are as follows. EA profoundly activates PI3K/Akt signalling, resulting in the inhibition of cerebral cell apoptosis in the ischaemic penumbra. At the same time, EA increases the expression of PI3K, p-Akt, p-Bad, and Bcl-2 at the protein level and increases the expression of Bcl-2 at the mRNA level. However, EA inhibits Bax and reduces the positive expression of caspase-3. The PI3K/Akt pathway plays a critical role in mediating the neuroprotective effects of EA treatment at Zusanli (ST36) and Quchi (LI11) after ischaemic stroke [21]. The ST36 acupoint suppresses the haemorrhage-induced increase in lesion size and apoptotic neuronal cell death in the striatum. Acupunctural treatment at the ST36 acupoint may aid in recovery following central nervous system sequelae following intracerebral haemorrhage [39]. EA treatment further increases the antioxidant enzyme activities in ischaemic-reperfused brain tissues, with a concomitant decrease in the extent of lipid peroxidation. EA treatment at Fengchi (GB20) reduces the extent of lipid peroxidation in ischaemic-reperfused rat brains, possibly by increasing the activities of SOD and GPx [40]. EA preconditioning and postconditioning could alleviate spinal cord I/R injury, which is partly mediated by autophagy upregulation-induced inhibition of apoptosis and neuroinflammation [41]. In addition, EA pretreatment significantly reduced neuronal apoptosis, preserved neuronal morphology, and inhibited caspase-3 activity in the hippocampal CA1 area caused by +Gz exposure. EA pretreatment also ameliorates learning and memory function in rats exposed to +Gz [42]. The severity of cerebral ischaemia–reperfusion is improved with EA treatment. Oxidative and inflammatory damage are also alleviated with EA intervention. EA can enhance mitochondrial respiratory chain enzyme activity, reduce mitochondrial damage, and inhibit neuronal apoptosis. It is assumed that 4-VO-induced cerebral ischaemia/reperfusion might be alleviated by EA through the inhibition of excessive autophagy in neurons in the reperfusion period [43].

The main points of the articles included in PLOS One are as follows. EA at acupoints 1 d after reperfusion effectively downregulates astrocytic S100B expression to provide neuroprotection against delayed infarct expansion by modulating p38 MAP kinase-mediated NF-kappa B expression. These effects subsequently reduce oxidative/nitrative stress and inhibit the TNF-alpha/TRADD/FADD/cleaved caspase-8/cleaved caspase-3 apoptotic pathway in the ischaemic cortical penumbra 7 d after reperfusion [44].

The main points of the articles included in Journal of Cerebral Blood Flow and Metabolism are as follows. The parasympathetic nervous system promotes both the functional benefits of EA and its effects in improving cerebral perfusion. EA reduces infarct volume and hinders apoptosis, neuronal and peripheral inflammation, and oxidative stress. EA results in less reduction in the mRNA level of choline acetyltransferase, five subtypes of muscarinic receptors, and a7nAChR, suggesting the inhibition of the impairment of the central cholinergic system. EA also activates the dorsal motor nucleus of the vagus, the largest source of parasympathetic preganglionic neurons in the lower brainstem (detected by c-fos immunohistochemistry), and PD suppresses these changes [45]. EA pretreatment results in increased ambient endocannabinoid (eCB) levels and subsequent activation of ischaemic penumbral astroglial cannabinoid type 1 receptors (CB1Rs), which leads to moderate upregulation of extracellular glutamate that protects neurons from cerebral ischaemic injury [46].

The main points of the articles included in Evidence-Based Complementary and Alternative Medicine are as follows. Electroacupuncture therapy for CCH may be mediated by the miR-137/NOX4 axis [47]. The alleviation of inadequate autophagy and apoptosis may be a key mechanism involved in the reflex regulation of EA at GV26 to treat cerebral ischaemia [48]. EA treatment improves learning and memory function in AD rat models partially by downregulating the Notch signalling pathway [49]. Both EA pretreatment and treatment significantly reduce infarct volumes, decrease brain water content, and alleviate neuronal injury in MCAO rats. EA exerts neuroprotection against I/R injury by improving neurological function, attenuating inflammatory cytokines, upregulating antioxidant systems, and reducing excitotoxicity [50]. EA stimulation at acupoints prior to ischaemia leads to neuroprotection and myocardial protection and induces rapid and delayed ischaemic tolerance. The protective mechanisms of EA pretreatment may involve a series of regulatory molecular pathways, including antioxidant activity enhancement, endocannabinoid system regulation, beta-adrenergic receptor involvement and its postreceptor signalling pathway, and apoptosis inhibition. EA pretreatment can be a new preventive strategy for patients with a high risk of ischaemia in the clinic [5].

**Figure 5:**
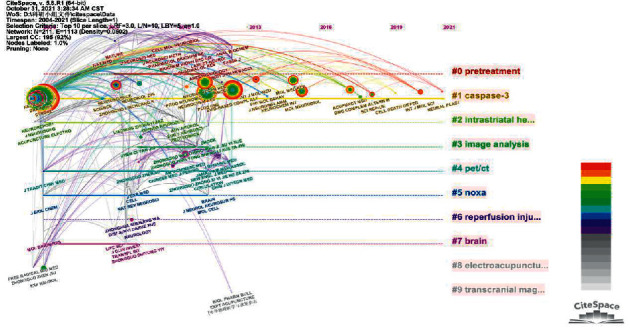
Keyword cluster map of major journals/topic cluster map of major journals.

Moreover, the research topics that journals focus on can also provide references for researchers to track research hotspots. In Figure 5, we cluster journals according to keywords/topics and add time coordinates. Our aim is to dynamically present important journals in various subfields of acupuncture and antiapoptosis research. The journals in the figure are divided into 9 clusters: #0 pretreatment, #1 caspase-3, #2 intrastriatal haemorrhage, #3 image analysis, #4 PET/CT, #5 noxa, #6 reperfusion injury, #7 brain, #8 electroacupuncture, and #9 transcranial magnetic stimulation.

The distance extended by the solid line in Figure 5 represents how long the study lasted. The position of the dot and the text indicates the time when the journal's research in the field appeared. We know some basic information from keyword clustering. Transcranial magnetic stimulation and PET/CT are important detection and analysis methods. Noxa and caspase-3 are important laboratory test indicators. Reperfusion injury is a common cause of nerve cell injury. EA and acupuncture pretreatment are the main intervention methods. The distance extended by the solid line in the figure represents how long the study lasted. The position of the dot and the text indicates the time when the journal's research in the field appeared.

### 3.6. Research Hotspot Analysis

The keywords in each paper can summarize the main idea of the relevant research. Therefore, the main research hotspots in the field can be displayed by analysing the keywords of the literature in acupuncture for the treatment of central nervous system cell apoptosis. The field of acupuncture for the treatment central nervous system cell apoptosis involves 838 keywords. There are 17 keywords with a frequency greater than 15 (Table 5).

These keywords are mostly related to central nervous system diseases, nerve cell apoptosis, and nerve cell protection. “Artery occlusion” and “rapid tolerance” have a high outbreak value, although their frequency of occurrence is not very high. This indicates that there are new research hotspots in artery occlusion and rapid dilatation. Therefore, the diseases involved in acupuncture-induced antiapoptosis of nerve cells include stroke and cerebral ischaemia, and their mechanisms are mostly related to oxidative stress, gene expression, and artery occlusion.

Due to the large amount of keyword data, artificial methods have been unable to carry out more in-depth analyses. We used the keyword clustering function of CiteSpace to cluster all the keywords. According to the degree of correlation between keywords, we clustered out a number of research topics. The obtained keywords can represent previous research hotspots in this field and provide references for predicting new research directions.

**Figure 6:**
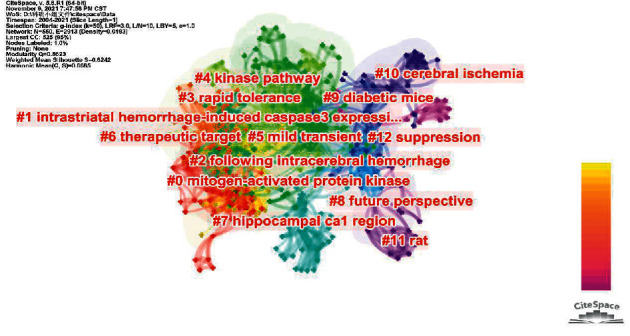
Keyword cluster map.

The keywords are divided into 13 clusters in Figure 6: #0 mitogen-activated protein kinase, #1 intrastriatal haemorrhage-induced caspase-3 expression, #2 following intracerebral haemorrhage, #3 rapid tolerance, #4 kinase pathway, #5 mild transient, #6 therapeutic target, #7 hippocampal CA1 region, #8 future perspective, #9 diabetic mice, #10 cerebral ischaemia, #11 rat, and #12 suppression. Each cluster word is preceded by a serial number from 0 to 12.

We found that the MAPK signalling pathway, caspase-3, and hippocampal CA1 area are still hotspots in the field of acupuncture for the treatment of central nervous system cell apoptosis. Cerebral haemorrhage and mild transient ischaemia may receive more attention than cerebral ischaemia in the future. Acupuncture preconditioning to improve the rapid tolerance of central nervous system cells to focal ischaemia has received attention for some time. In the selection of animal models, researchers have begun to pay attention to animal models with underlying diseases, such as diabetic mice.

## 4. Conclusions

This article introduces, for the first time, a qualitative and quantitative review of 18 years (2004–2021) of research on the antineuronal apoptosis induced by acupuncture and moxibustion. The main content includes the temporal and spatial distribution of research resources, research teams with outstanding contributions, and research hotspots. We have drawn three main conclusions, as follows.

**Figure 7:**
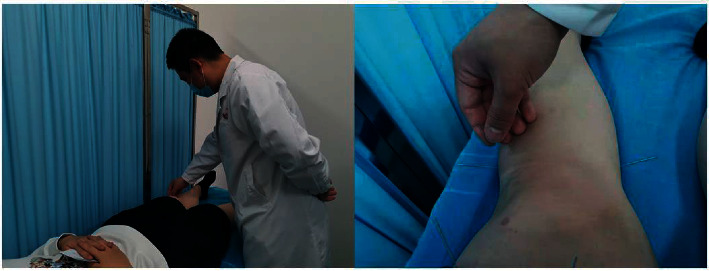
A patient with cerebral ischaemic injury is receiving acupuncture treatment.

  First, the field of acupuncture and antineuronal apoptosis has attracted increasing attention. The annual number of articles presented an increasing trend. Especially after 2013, France, Canada, Britain, Egypt, and Iran participated in research on the acupuncture-induced antiapoptosis of nerve cells. The number of articles published in this field has increased significantly. To date, China and America have taken the leading position in the research field of acupuncture and antineuronal apoptosis. The research institutes with much research output are all located in China. The United States engages in extensive international cooperation in this area.
  Second, 5 stable and high-yielding research teams have been formed in the research field of acupuncture and antineuronal apoptosis. Their main research directions are EA pretreatment for central nervous system cell apoptosis, acupuncture for antineuronal apoptosis in vascular dementia, EA regulation of related signalling pathways, EA regulation of nerve cell apoptosis and autophagy after stroke, and EA regulation of the MAPK signalling pathway. The more widely the researchers and teams collaborate, the more research results and the better sustainability of the research are. Feng Zhang's team tracks popular issues and has published numerous papers in recent years. The team is expected to make great contributions to the acupuncture and antineuronal apoptosis field.
  Third, the MAPK signalling pathway, caspase-3, and hippocampal CA1 areas are still hotspots in the field of acupuncture for the treatment of central nervous system cell apoptosis. Cerebral ischaemia–reperfusion injury is the most concerning pathological state. However, cerebral haemorrhage and mild transient ischaemia may receive more attention than cerebral ischaemia in the future. More detection methods will be applied to research in this field, such as transcranial magnetic stimulation and PET/CT. In addition, researchers have begun to pay attention to animal models with underlying diseases, such as diabetic mice.
  In this study, the hotspots and frontiers of acupuncture for the treatment of central nervous system cell apoptosis were the focus of our attention. Due to article length limitations, we are not able to illustrate all the results and details of our findings. Even so, this paper still has important reference value.
  In the future, we will apply a variety of data visualization tools to analyse the relevant data from multiple databases to overcome the limitations of CiteSpace software. In addition, we will pay attention to differences in the microstructure of documents to discover landmark research results in the field and show the correlation between different research topics.
  Finally, we have supplemented 2 photos related to acupuncture treatment of diseases (Figure 7).

## Figures and Tables

**Table 1:** All countries and regions involved in the field of acupuncture and antineuronal apoptosis.

Rank	Frequency	Centrality	Countries and regions	Year
1	137	0.5	China	2004
2	10	0	Taiwan	2007
3	6	0	South Korea	2004
4	4	0.29	USA	2004
5	2	0	Canada	2014
6	2	0	England	2018
7	2	0	France	2013
8	1	0	Iran	2019
9	1	0	Egypt	2015

**Table 2:** Main authors' cooperation networks and cooperation network information form.

Rank	Representative authors	Main research areas
1	Qiang Wang	Study on the mechanism of EA pretreatment for the treatment of nerve cell apoptosis.
2	Cunzhi Liu	Acupuncture improves cognitive impairment in rats with vascular dementia through antioxidant and antiapoptotic effects.
3	Lidian Chen	EA regulates related signal pathways to play a neuroprotective role.
4	Feng Zhang	EA regulates nerve cell apoptosis and autophagy after stroke by regulating related signal pathways.
5	Chunxiao Wu	EA inhibits neuronal apoptosis in rats with cerebral ischaemia and reperfusion by regulating the MAPK signalling pathway.

**Table 3:** Authors with 5 or more publications.

Rank	Frequency	Burst	Centrality	Author	Year
1	16	3.95	0.02	Qiang Wang	2009
2	13	3.51	0	Lize Xiong	2009
3	7		0	Lidian Chen	2012
4	7		0.01	Cunzhi Liu	2009
5	7		0.01	Feng Wang	2011
6	6	3.06	0	Xin Li	2011
7	6		0	Feng Zhang	2014
8	6		0	Jing Tao	2012
	5		0	Fang Dong	2018
	5		0	Chingliang Hsieh	2007
	5		0	Jia Huang	2012
	5		0	Ying Xing	2018
	5		0	Jun Peng	2012
	5		0	Xuerui Wang	2016

**Table 4:** Journals cited more than 50 times.

Rank	Frequency	Journal	Year
1	110	Stroke	2004
2	94	Brain Res	2004
3	91	Neurosci Lett	2004
4	77	PLOS One	2011
5	74	J Cerebr Blood F Met	2004
6	69	J Neurosci	2004
7	68	Evid-Based Compl Alt	2012
8	58	Neural Regen Res	2011
9	54	Neuroscience	2007
10	50	Mol Neurobiol	2013

**Table 5:** Keywords that appear more than 15 times.

Rank	Freq	Keyword	Burst
1	84	Apoptosis	
2	83	Electroacupuncture	
3	67	Acupuncture	
4	44	Expression	
5	39	Stroke	
6	39	Activation	
7	28	Cerebral ischaemia	
8	27	Rat	
9	25	Brain	
10	23	Artery occlusion	3.38
11	21	Mechanism	
12	21	Focal cerebral ischaemia	
13	20	Neuroprotection	
14	19	Oxidative stress	
15	19	Ischaemia	
16	17	Rapid tolerance	3.13
17	17	Model	

## Data Availability

The data used to support the findings of this study are included within the article.
